# Dangerous Liaisons: Interactions of *Cryptococcus neoformans* with Host Phagocytes

**DOI:** 10.3390/pathogens9110891

**Published:** 2020-10-27

**Authors:** Elizabeth A. Gaylord, Hau Lam Choy, Tamara L. Doering

**Affiliations:** Department of Molecular Microbiology, Washington University School of Medicine, Saint Louis, MO 63110, USA; egaylord@wustl.edu (E.A.G.); choyk@wustl.edu (H.L.C.)

**Keywords:** *Cryptococcus neoformans*, host-pathogen interactions, non-lytic exocytosis, pathogenesis, phagocytes, Trojan horse transit

## Abstract

*Cryptococcus neoformans* is an opportunistic fungal pathogen and a leading cause of death in immunocompromised individuals. The interactions of this yeast with host phagocytes are critical to disease outcome, and *C. neoformans* is equipped with an array of factors to modulate these processes. Cryptococcal infection begins with the deposition of infectious particles into the lungs, where the fungal cells deploy various antiphagocytic factors to resist internalization by host cells. If the cryptococci are still engulfed, they can survive and proliferate within host cells by modulating the phagolysosome environment in which they reside. Lastly, cryptococcal cells may escape from phagocytes by host cell lysis, nonlytic exocytosis, or lateral cell-to-cell transfer. The interactions between *C. neoformans* and host phagocytes also influence the dissemination of this pathogen to the brain, where it may cross the blood-brain barrier and cause an often-fatal meningoencephalitis. In this review, we highlight key cryptococcal factors involved in various stages of cryptococcal-host interaction and pathogenesis.

## 1. Introduction

*Cryptococcus neoformans* is an important fungal pathogen that primarily infects immunocompromised individuals, particularly those with HIV. Worldwide cases of cryptococcal meningitis decreased from approximately 1 million in 2008 to one quarter of this number in 2014, in part due to increased implementation of antiretroviral therapies [1,2,3]. However, the unique biology of this organism continues to make treatment of cryptococcal infections a challenge. This review will discuss the multifaceted strategies that allow *C. neoformans* to be a successful pathogen, with a focus on its unique interactions with host phagocytes. 

**Figure 1:**
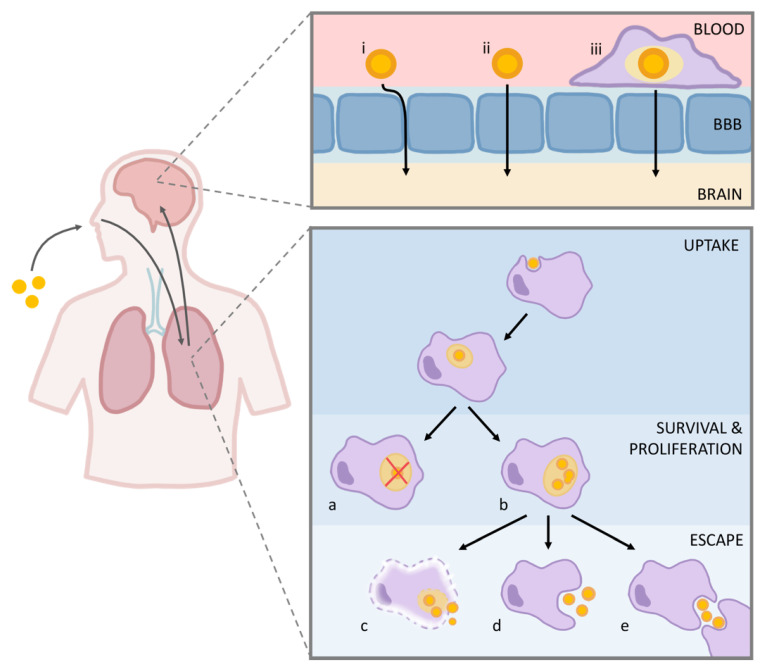
*C. neoformans* interactions with host cells. Left, inhalation of infectious particles and dissemination to the brain. Right, events discussed in the text: Top, blood-brain barrier (BBB) traversal by *C. neoformans* may be paracellular (i), transcellular (ii), or via Trojan horse transit (iii). Bottom, possible fates of *C. neoformans* after engulfment include fungal clearance (a), proliferation (b), lytic escape (c), nonlytic exocytosis (d), and cell-to-cell transfer (e).

Cryptococcal infection begins with the inhalation of spores or desiccated yeasts and their deposition into the alveoli, where they may be internalized by host phagocytes. In immunocompetent individuals, most of the fungal cells are cleared by the immune system, although a subset can remain latent in the lung. In immunocompromised individuals, cryptococci survive and proliferate within host phagocytic cells and may later escape via various strategies. Free yeast cells or infected phagocytes may also subsequently disseminate to other body compartments, traverse the blood-brain barrier, and enter the central nervous system, which can lead to life-threatening fungal meningitis (Figure 1). The following sections highlight key factors in each stage of cryptococcal pathogenesis.

## 2. Entry

*C. neoformans* infection begins when an infectious propagule is inhaled and deposited in the lung. Interestingly, both spores and desiccated yeast cells have the aerodynamic properties necessary to reach the alveoli [4]. Spores have a thick protective coat and are readily engulfed by macrophages, inside which they germinate, consistent with a role in initiating infection [5]. However, the mating and fruiting structures that produce spores have not been observed in nature, suggesting that desiccated yeasts may be the more common type of infectious particle, even though they lack the resilience of spores [5]. Historically, most animal models of cryptococcosis have used cultured yeast cells. Notably, inoculation with spores from some strains that appear to be avirulent as yeasts can cause fatal disease in mice [6]. Thus, spores may have an underestimated role in pathogenesis. The question of whether spores or yeasts are the dominant particle in natural infection remains open.

## 3. Uptake

Following entry and deposition into the lungs, most *C. neoformans* cells are internalized by phagocytes [7], despite their utilization of various antiphagocytic strategies (discussed below). The outcome of this interaction can range from fungal cell death to dissemination of the infection. Diverse cryptococcal factors influence this process, as discussed below.

### 3.1. Capsule as an Antiphagocytic Factor

The characteristic polysaccharide capsule is a major virulence factor of *C. neoformans*. This structure is composed of two large repeating polymers, glucuronoxylomannan (GXM) and glucuronoxylomannogalactan (GXMGal), in addition to trace mannoproteins [8,9]. GXM features a mannose backbone with xylosyl and glucuronyl side groups, while GXMGal consists of a galactose backbone with galactomannan side chains that are modified with xylose, glucuronic acid, and galactofuranose [10]. Although the capsule itself is weakly immunogenic, it masks the underlying cell wall, which is composed of multiple highly immunogenic components that are readily recognized by the innate immune system, such as β-glucans, ⍺-mannans, and chitin [11,12,13].

The capsule also physically inhibits phagocytosis by increasing cell size. Upon entry of *C. neoformans* into the host environment, capsule thickness enlarges dramatically; as capsule size increases, the efficiency of phagocytosis decreases [14]. Accordingly, acapsular strains are more avidly engulfed than normal encapsulated strains [15,16], although the association of isolated capsule polysaccharides with their surfaces has been shown to help them resist phagocytosis [17]. Mutant strains with altered or compromised capsule structures, such as those deficient in capsule synthesis, often exhibit increased uptake compared to wild-type cells [18,19,20,21,22], suggesting that specific capsule architecture is important for protection against phagocytosis.

### 3.2. Capsule-Independent Evasion of Phagocytosis

While capsule is a key factor in cryptococcal interactions with phagocytic cells, *C. neoformans* also employs a variety of other tools to avoid engulfment. One well-characterized example is antiphagocytic protein 1 (App1), a secreted protein that can be detected in the serum of infected individuals [23]. This protein, which also localizes to the cryptococcal cell wall, binds to complement receptor 3 (CR3) on the surface of monocytes, macrophages, and dendritic cells [24,25]. This association competitively inhibits the binding of serum-opsonized cryptococci by CR3, which normally mediates their internalization, and thereby reduces fungal engulfment [24]. Loss of App1 results in increased host phagocytosis of cryptococci, without affecting capsule formation. Interestingly, *APP1* deletion results in attenuated virulence in an A/Jcr murine infection model but increased virulence in mice that lack T and NK cells [23]. These results highlight the complex role of phagocytosis in cryptococcal infection. Additionally, Ghaffar et al. reported that the presence of App1 increases cryptococcal susceptibility to the common antifungals fluconazole and amphotericin B, both of which target fungal lipids [26]. This suggests that App1 has undiscovered functions in cryptococcal biology, in addition to its role in avoiding phagocytosis.

Other proteins involved in interactions of *C. neoformans* with host phagocytic cells have been identified through various genetic screens [27,28,29,30]. It is often difficult to untangle the functions of such proteins because they may affect multiple cell attributes that influence uptake, including capsule, cell wall, and virulence factor production. The transcription factor Gat201 is one such case. Deletion of *GAT201* yields cells that are hypocapsular and exhibit high levels of phagocytosis even in the absence of opsonization. However, the increased uptake of these mutant cells is not entirely due to their small capsule size, suggesting that Gat201 mediates both capsule-dependent and -independent evasion of phagocytosis [27]. The capsule-independent anti-phagocytic effects of Gat201 have been attributed to its regulation of two other proteins, Gat204 and Blp1 [30]. 

Cell size is another property that *C. neoformans* modulates to evade phagocytosis. Within 24 h of lung infection, normal cryptococcal cells, typically 5–7 μm in diameter, can form ‘titan’ cells that range from 10–100 μm in width. These massive cells resist uptake by host phagocytes [31,32,33] and are associated with high levels of lung escape, dissemination, and BBB crossing [34,35]. Titan cells also confer protection from phagocytosis on their normal-sized progeny, through a mechanism that is not yet understood [36]. Titan cells differ from normal-sized cells in their ploidy, cell wall composition, and capsule thickness. With the recent development of multiple protocols to induce titan cell formation in vitro, the field is poised to investigate their unique biology and the factors that govern their interactions with host cells [35,37,38,39].

Within the population of cryptococci in the lung, cells vary in age in addition to size. Older cells are more resistant to phagocytosis than younger cells [40]. This could potentially involve many factors that influence uptake, including age-dependent cell wall modifications [41] and expression of antiphagocytic factors like App1 [40].

Although *C. neoformans* employs numerous antiphagocytic mechanisms, most cryptococci are nonetheless successfully engulfed by host cells during infection, via opsonization or direct interaction of polysaccharide fibers with phagocyte receptors [7,42]. Once phagocytosed, fungal cells can survive and proliferate within macrophages. This raises the question of why *C. neoformans* engages multiple mechanisms to prevent uptake if it is capable of surviving within phagocytes. One possibility is that antiphagocytic factors enable fungal persistence at the primary infection site. Uptake avoidance may also give *C. neoformans* time to prepare for its subsequent survival and proliferation within phagocytes. Overall, the balance of evasion and uptake, and how each benefits the pathogen and the host, remain to be elucidated. 

## 4. Survival and Proliferation

After phagocytosis, the macrophage undergoes phagosome maturation, during which the *C. neoformans*-containing vacuole becomes an increasingly antimicrobial environment. This process includes the initiation of a respiratory burst, the production of a cocktail of antimicrobial enzymes, and the acidification of the phagosome. Acidification allows the phagosome to fuse with the lysosome, forming a phagolysosome that contains a potentially lethal combination of free radicals and acid-dependent degradative enzymes [43].

In 1973, Diamond and Bennett first discussed the potential advantage of host phagocytosis for fungal cells, when they observed that *C. neoformans* replicated faster within human macrophages than extracellularly [44]. Notably, *C. neoformans* may manipulate phagosome maturation to enhance its own intracellular survival and proliferation by inducing membrane damage, preventing complete phagosome acidification, and impairing host antimicrobial activities. Unlike the defense strategies of some other microbes [45,46,47], *C. neoformans* does not impair phagolysosome fusion [7,48,49,50]. However, it does damage the phagolysosome membrane, introducing discontinuities that prevent the organelle from becoming acidified [7,49,50,51,52,53]. This damage begins within one hour of fungal engulfment [53] and the amount of damage correlates with cryptococcal replication [51]. To address cause-and-effect, Davis et al. induced lysosome damage using photosensitizers and observed increased *C. neoformans* replication [51]; this suggests that phagolysosome permeabilization promotes cryptococcal survival and proliferation. 

The cryptococcal mechanism of membrane damage is still unknown, but it may be mechanical (due to cell proliferation or enlarging capsules) or enzymatic (due to secreted proteins). Supporting the latter, mutants defective in phospholipase B1 grow poorly inside macrophages compared to wild-type cells and induce less phagolysosomal membrane damage [52,54]. In addition, the virulence factor urease reduces membrane permeabilization, as macrophages containing urease-positive cryptococci sustained fewer phagolysosomal discontinuities compared to those containing urease-negative cryptococci [55]. Permeabilizing phagolysosome membranes may enable *C. neoformans* to escape antimicrobial factors without losing its intracellular niche; it may also provide an entry route for nutrients in the host cell cytosol that are otherwise inaccessible [53].

Phagosome acidification creates an optimal environment for the activities of many antimicrobial enzymes; *C. neoformans* apparently impedes this process to its own advantage [48,56,57]. De Leon-Rodriguez et al. showed that while phagosomes that engulf inert beads reach a pH of 4.2 within 3 h, phagosomes that engulf cryptococci (live or heat-killed) only reach a pH of approximately 4.8 in the same period, a value closer to the optimum for cryptococcal growth [57]. How *C. neoformans* accomplishes this remains to be elucidated but is likely to be multifactorial. The ABC transporter Afr1 may play a role, since cells that overexpress it reduce phagosome acidification to a greater degree than wild-type [58]. Capsule may also inhibit pH modulation by buffering phagosome acidification with its abundant glucuronic acid. Consistent with this idea, cells with more capsule showed greater influence on phagosome pH [57]. 

Beyond inducing host membrane damage and preventing phagosome acidification, cryptococci enhance their intracellular survival by thwarting other host antimicrobial activities. For example, the capsule protects *C. neoformans* from macrophage-mediated killing in several ways. For example, it inhibits macrophage nitric oxide induction, possibly by preventing the production of required signaling molecules such as TNFα [59]. Capsular GXM binds to macrophage CD14 and toll-like receptors 2 and 4, which leads to translocation of NF-*κ*B to the nucleus. However, there is no subsequent TNFα secretion, which is required for clearance of cryptococcal infections [13,60,61,62,63]. This may be because GXM does not stimulate mitogen-activated protein kinase (MAPK) pathways, which are critical for TNFα induction [63]. The capsule also provides resistance against reactive oxygen species (ROS) and free radicals encountered within the macrophage [62,63,64,65]. Finally, although the specific mechanisms are not known, cryptococci alter calcium flux, which is critical for proper phagosome maturation, and hinder the activity of cathepsin L, an antimicrobial protease [56]. With multiple tools to hinder host microbicidal processes, *C. neoformans* is well-equipped to survive and proliferate in the intracellular environment. 

## 5. Escape

While many pathogens captured by macrophages are degraded in the phagosome, *C. neoformans* uses at least three mechanisms to escape this fate: lytic exocytosis, which destroys the host cell, nonlytic expulsion, which leaves the host cell intact, and cell-to-cell transfer between macrophages. These strategies, which are discussed below, promote cryptococcal survival and ultimately dissemination in human hosts.

### 5.1. Lytic Escape

Phagocyte lysis has been observed in cryptococcal infections [66,67], even though *C. neoformans* does not express the pore-forming proteins used by other pathogens for this process [68]. It may be that rapid proliferation of intracellular cryptococci or accumulation of shed capsular polysaccharides causes mechanical rupture of the host cell [68], although neither possibility has been directly tested. Another possibility is that glycosylated mannoproteins in the cell wall induce host cell lysis, as acapsular but not encapsulated heat-killed cells trigger macrophage lysis; tellingly, glycosidase treatment of acapsular cells reduces this lytic capability [67]. 

### 5.2. Non-Lytic Exocytosis

In some cases, cryptococci escape from macrophages via non-lytic exocytosis (NLE), also known as phagosomal extrusion or vomocytosis. This process, which leaves the host cell viable, occurs when the membrane of the phagolysosome fuses with the plasma membrane to release cryptococcal cells extracellularly [50,53,68,69,70]. Time-lapse microscopy studies show that NLE is a rapid process lasting from 4–12 minutes [71] and that it occurs within 2–8 h after macrophage infection [50,69,71]. NLE has been primarily studied in *C. neoformans*, where it is the most common exit strategy [69], but also occurs following engulfment of other fungi (*Cryptococcus gattii, Candida albicans*) and bacteria (*Chlamydia* spp., and *Orientia tsutsugamushi*) [72,73,74,75]. The molecular mechanisms of NLE initiation and regulation are not known. 

NLE requires viable cryptococci, as both heat-killed fungal cells and beads can be engulfed by macrophages but neither escape via NLE [50,69]. Acapsular strains are also deficient in NLE; this may be because capsule promotes NLE, or because acapsular strains survive poorly in macrophages [50]. A recent study showed that laccase, an enzyme involved in cell melanization and stress protection, also affects NLE in a manner independent of its other roles [76]. Phospholipase B1 is another virulence factor implicated in this process, as mutants deficient in Plb1 secretion are impaired in NLE [77]. This lipase liberates macrophage-derived fatty acids for use in cryptococcal eicosanoid synthesis [78,79]; it is possible that its function in lipid metabolism contributes to cryptococcal NLE. Consistent with this idea, NLE is enhanced by the addition of oleic acid to macrophage cultures, which increases both macrophage and cryptococcal lipid droplets [80]. 

Host components also modulate NLE. After *C. neoformans* damages the phagolysosome, the host cell undergoes repeated cycles of actin polymerization and disassembly (actin flashes) around the organelle [50,53,69]. As actin flashes increase, the frequency of cryptococcal expulsion decreases [53], suggesting that this host process counteracts cryptococcal NLE. The MEK5/ERK5 pathway, which is important for cell growth, survival, and proliferation, is also involved in NLE regulation. Gilbert et al. showed that stimulation of the MEK5/ERK5 pathway inhibited NLE, while disruption of this pathway significantly increased expulsion rates [81]. Lastly and unsurprisingly, the immune state of the host affects the ability of cryptococcal cells to undergo NLE. For example, coinfection of macrophages with HIV or measles virus enhanced NLE without altering fungal uptake or intracellular proliferation [82]. The authors of this study proposed that the host response to viral infection, rather than the virulence of the virus, stimulates fungal cell expulsion via induction of type-I interferon signaling [82]. This effect of viral coinfection on NLE was not observed in *C. albicans* infection [82].

The non-lytic escape of cryptococcal cells from host phagocytes has important implications for disease progression. The occurrence of NLE varies significantly depending on both the host cell type and the *Cryptococcus* species (which may serve to explain the differing pathogenicity between *C. neoformans* and its non-opportunistic cousin *C. gattii*) [50,83]. Furthermore, depending on the timing and context of NLE, it may either inhibit or accelerate disease. For example, if expulsion occurs within the lungs, it may benefit the host as a mechanism to “reset” macrophages if killing is unsuccessful [82]. This could also contain the infection in the lungs, as macrophages would not convey the fungal cells to other body compartments. If the cryptococci-containing macrophages are outside of the lung, NLE may be deleterious to the host, as discussed below in the context of dissemination. 

### 5.3. Cell-to-Cell Transfer

A third cryptococcal escape mechanism is movement between phagocytes, although this occurs with only 2% of infected macrophages [84,85]. Similar to NLE, this process requires viable cryptococci; it is not observed with heat-killed *C. neoformans* [84,86]. During this lateral transfer, the donor and acceptor macrophages must be in contact, with the acceptor cell extending cytoplasmic projections towards the donor macrophage in an actin-dependent manner [84,85]. Although this movement has been categorized as a distinct strategy for cryptococcal escape, some researchers argue that it simply represents immediate engulfment of expelled fungal cells by a neighboring phagocyte [71,86]. In either case, lateral transfer allows intracellular cryptococci to avoid further exposure to the host immune system, which likely favors pathogen survival and dissemination [84,85,86].

## 6. Dissemination

Cryptococci disseminate from the lung either within host phagocytes or as free fungi (reviewed in detail by Denham and Brown, 2018) [87], although the precise contribution of each mechanism is unknown. Various fungal proteins promote this process. Cells lacking the glucosylceramide synthase Gcs1 are unable to grow in alkaline environments, including the lungs and bloodstream [88]. Additionally, these cells are avirulent in an intranasal infection model but can cause fatal disease when the lung is bypassed by intravenous inoculation [89]. These observations are consistent with a role for plasma membrane glucosylceramides in cryptococcal dissemination. Phospholipase B1 mutants demonstrate delayed dissemination in a murine endotracheal infection model, suggesting a role for this enzyme in bloodstream entry in addition to its functions in phagosome survival and escape mentioned above [90].

*C. neoformans* disseminates to multiple organs, including the kidneys, liver, and spleen, but it is specifically dissemination to the central nervous system (CNS) that leads to death in the majority of patients [91,92]. The level of neurological involvement is one feature that distinguishes *C. neoformans* and *C. gattii* infections [93,94,95]. Cryptococcal neurotropism is influenced by the transcription factor Hob1, which was identified by Lee et al. as a ‘master regulator’ of brain infection in *C. neoformans,* but not in *C. gattii* [96]. Comparisons between these species may be useful in understanding the greater predilection of *C. neoformans* for the CNS.

To infect the CNS, cryptococcal cells must cross the blood-brain barrier (BBB), which normally restricts the passage of cells and solutes between the circulating blood and the brain [97]. Three potential methods of cryptococcal migration across the BBB have been proposed: transcellular migration through endothelial cells, paracellular migration between endothelial cells, and “Trojan horse” passage (transcellular or paracellular) inside phagocytic cells. These models, summarized briefly below, are not mutually exclusive, although the relative impact of each on CNS infection is still unknown. 

Two models of BBB transit are independent of phagocytes. In the transcellular model cryptococci pass directly through endothelial cells of the BBB, likely entering the cells via endocytosis at host cell lipid rafts [88,89]. Paracellular crossing occurs when fungal cells adhere to BBB endothelia and induce cytoskeletal rearrangements, potentially modifying the tight junctions between host endothelial cells to facilitate passage [90,91].

The third model of BBB traversal is Trojan horse passage inside of a host phagocyte. Several early studies suggested that phagocytic cells promote cryptococcal dissemination to the brain [89,90,98,99]. These included the observation that murine infection with cryptococcal cells within macrophages yields higher fungal burden in the brain than infection with free cells [99]. The Trojan horse mechanism is supported by recent studies that used trans-well models and live imaging to directly demonstrate the ability of engulfed cryptococci to cross an endothelial cell monolayer [100,101]. Interestingly, some mutants that lack the ability to cross the BBB alone are capable of Trojan horse transit, suggesting that traversal of the BBB may employ multiple strategies [101].

While many questions remain about how *C. neoformans* crosses the BBB, a variety of cryptococcal factors have been implicated in this transition. Cryptococci are sequestered in brain microcapillaries prior to traversal, a process which requires the enzyme urease [102,103]. Hyaluronic acid synthesis by the glycosyltransferase Cps1 helps the yeast adhere to brain endothelial cells prior to BBB crossing [104]. Phospholipase B1 activity appears to be important for adhering to and crossing the BBB endothelial layer, along with its roles in earlier stages of pathogenesis mentioned above [105,106,107]. Also implicated in BBB traversal are the inositol transporters Itr1a and Itr3c, which may aid in nutrient acquisition in the inositol-rich brain environment. Compared to parental strains, cells lacking both of these proteins shed less capsular material and (perhaps as a result) induce a stronger host immune response in the brain [108,109]. Finally, cryptococci that lack the multidrug resistance protein Fnx1 can reach the BBB in a zebrafish model of infection but are incapable of crossing into the CNS [110,111].

The secreted metalloprotease Mpr1 is required for cryptococcal BBB crossing and expression of this cryptococcal gene in *Saccharomyces cerevisiae* enables the model yeast to migrate across the BBB in a hyaluronic acid-independent manner [112,113]. Mpr1 also interacts with the host surface protein AnnexinA2 (AnxA2), via an unknown mechanism, which leads to this transcytosis [114]. Interestingly, inhibition of AnxA2 with antibodies prevents transcellular BBB crossing of Mpr1-expressing *S. cerevisiae* in vitro, although the early steps of binding and entry of endothelial cells occur [114].

Cryptococcal-derived microvesicles (CnMVs), which are reported to contain capsular material, may also promote BBB traversal [115,116]. Huang et al. demonstrated that purified CnMVs increased BBB crossing by *C. neoformans* in an in vitro model and raised brain fungal burdens when included in tail vein infections. The authors speculated that CnMVs have yet undefined roles in promoting cryptococcal virulence [117].

Once it has crossed the BBB, *C. neoformans* proliferates within the CNS, where it causes a frequently fatal meningoencephalitis [91,118]. The melanin-producing enzyme laccase, mentioned above, is also required for survival in this environment [119,120]. Recently, Lee et al. used direct intracerebroventricular infection to identify two transcription factors and six kinases needed for survival in the brain parenchyma [96].

## 7. Conclusions and Future Directions

**Figure 2:**
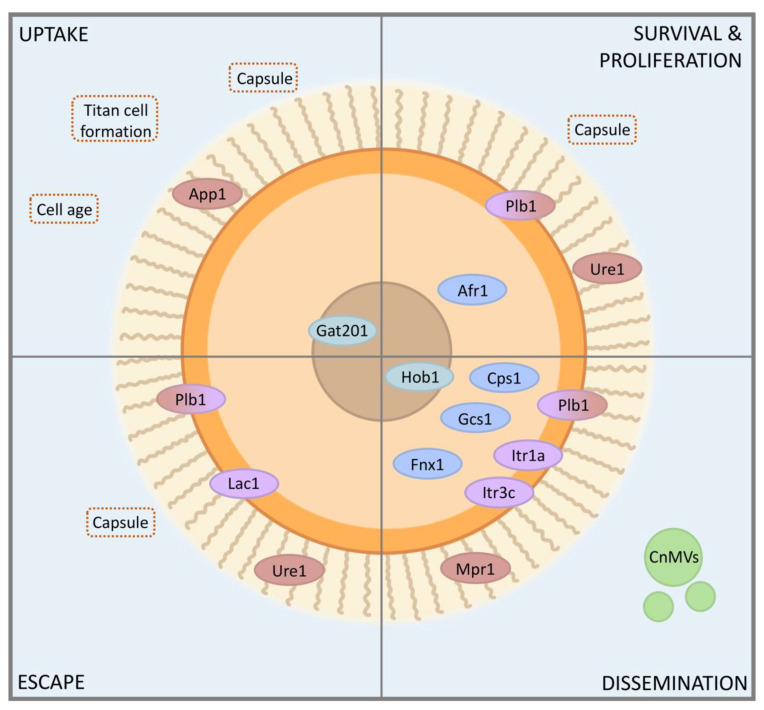
Schematic of cryptococcal features discussed in the text that influence interactions with host cells. See text for details. Concentric cellular regions: brown, nucleus; pale orange, cytosol; bright orange, cell wall; beige, capsule. Labeled ovals: light blue, transcription factors; dark blue, intracellular proteins; purple, plasma membrane/cell wall proteins; maroon, secreted proteins. CnMVs, cryptococcal microvesicles.

The tension between Cryptococcus’ antiphagocytic arsenal and its ability to leverage phagocytosis as a means of dissemination culminates in the pivotal moment of encounter between *C. neoformans* and host phagocytes. This interaction leads to fates which range from clearance of fungal cells to disseminated infection. While *C. neoformans* has an arsenal of factors that modulate each stage of infection (Figure 2), the complex nature of fungal-host interactions is still not fully understood. Some of the outstanding questions in the field are summarized in Box 1. Addressing these topics to better understand how *C. neoformans* interacts with the host, and the roles of key fungal factors in these processes, will aid future efforts to combat this devastating pathogen.

Box 1 **Key Open Questions in *C. neoformans* pathogenesis.**

Entry:

- What are the relative contributions of spores and desiccated yeasts in natural infection?
- What are the differences between the pathogenesis of spore and yeast infections?

Uptake:

- Are there multiple capsule-independent antiphagocytic factors and, if so, how do they act?
- How do titan cells benefit their progeny?
- What is the balance between avoidance and occurrence of phagocytosis, and how does each outcome affect the pathogen and host?

Survival/Proliferation:

- How does *C. neoformans* damage phagolysosomal membranes?
- How does *C. neoformans* inhibit phagosome acidification?
- What determines the fate of engulfed *C. neoformans*?

Escape:

- What controls the mode of *C. neoformans* escape from phagocytes?
- How are various escape strategies induced and regulated?
- How does NLE differ between pathogens?

Dissemination:

- What are the relative contributions of free and engulfed cryptococci to dissemination?
- What is the balance of transcellular migration, paracellular migration, and “Trojan horse” transit in brain entry?
- How do CnMVs promote CNS invasion?
